# 2-(2-Chloro­phen­yl)-2,3-dihydro­quinazolin-4(1*H*)-one

**DOI:** 10.1107/S1600536809031328

**Published:** 2009-08-15

**Authors:** Ming-Jian Li, Chang-Jun Feng

**Affiliations:** a School of Chemistry & Chemical Engineering, Xuzhou Institute of Technology, Xuzhou Jiangsu 221008, People’s Republic of China

## Abstract

The title compound, C_14_H_11_ClN_2_O, was synthesized by the reaction of 2-chloro­benzaldehyde and 2-amino­benzamide in an ionic liquid. The pyrimidine ring adopts a skew-boat conformation and the two benzene rings make a dihedral angle of 87.1 (1)°. In the crystal, N—H⋯O and C—H⋯N hydrogen bonding links the mol­ecules along *b*.

## Related literature

For quinazoline derivatives as anti­tumor agents, see: Feng *et al.* (2006 ▶); Keenan & Shakespear (2004 ▶); Mikiciuk-Olasik *et al.* (2004 ▶). For the biological activity of quinazoline derivatives, see: Bedi *et al.* (2004 ▶); Lin *et al.* (2006 ▶); Saleh *et al.* (2004 ▶).
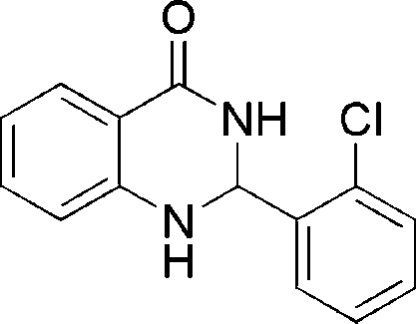


## Experimental

### 

#### Crystal data

C_14_H_11_ClN_2_O
*M* *_r_* = 258.70
Triclinic, 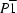

*a* = 6.9900 (1) Å
*b* = 8.7488 (2) Å
*c* = 10.4756 (2) Å
α = 100.639 (1)°
β = 92.726 (1)°
γ = 101.786 (1)°
*V* = 613.91 (2) Å^3^
*Z* = 2
Mo *K*α radiation
μ = 0.30 mm^−1^
*T* = 296 K
0.47 × 0.15 × 0.15 mm

#### Data collection

Bruker SMART CCD area-detector diffractometer
Absorption correction: multi-scan (Jacobson, 1998 ▶) *T*_min_ = 0.901, *T*_max_ = 0.950
8018 measured reflections
2204 independent reflections
2029 reflections with *I* > 2σ(*I*)
*R*_int_ = 0.019

#### Refinement

*R*[*F* ^2^ > 2σ(*F* ^2^)] = 0.030
*wR*(*F* ^2^) = 0.082
*S* = 1.07
2204 reflections
176 parameters
H atoms treated by a mixture of independent and constrained refinement
Δρ_max_ = 0.20 e Å^−3^
Δρ_min_ = −0.19 e Å^−3^

### 

Data collection: *SMART* (Bruker, 2001 ▶); cell refinement: *SAINT* (Bruker, 2001 ▶); data reduction: *SAINT*; program(s) used to solve structure: *SHELXS97* (Sheldrick, 2008 ▶); program(s) used to refine structure: *SHELXL97* (Sheldrick, 2008 ▶); molecular graphics: *SHELXTL* (Sheldrick, 2008 ▶); software used to prepare material for publication: *SHELXTL*.

## Supplementary Material

Crystal structure: contains datablocks global, I. DOI: 10.1107/S1600536809031328/pb2004sup1.cif

Structure factors: contains datablocks I. DOI: 10.1107/S1600536809031328/pb2004Isup2.hkl

Additional supplementary materials:  crystallographic information; 3D view; checkCIF report

## Figures and Tables

**Table 1:** Hydrogen-bond geometry (Å, °)

*D*—H⋯*A*	*D*—H	H⋯*A*	*D*⋯*A*	*D*—H⋯*A*
N2—H2*A*⋯O1^i^	0.831 (16)	2.461 (16)	3.1847 (16)	146.2 (14)
N1—H1*A*⋯O1^ii^	0.824 (18)	2.103 (18)	2.9146 (16)	168.4 (16)
C1—H1*B*⋯N2^iii^	0.948 (14)	2.635 (14)	3.4369 (17)	142.6 (11)

Symmetry codes: (i) 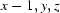; (ii) 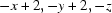; (iii) 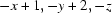.

**Fig. 1.**
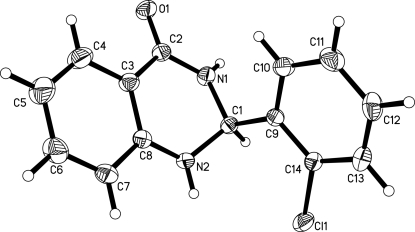
The molecular structure drawing for (I) showing 50% probability of displacement ellipsoids and the atom-numbering scheme.

**Fig. 2.**
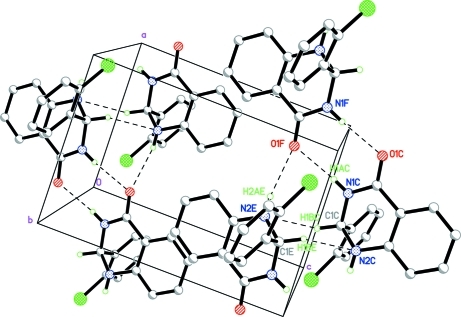
The molecular packing diagramshowing the hydrogen-bonding network in the crystal for (I).

## supplementary crystallographic information

### Comment

Quinazoline derivatives are well known compounds as antitumor agents (Feng *et al.*, 2006; Keenan *et al.*, 2004; Mikiciuk-Olasik *et al.*, 2004). In addition, it was reported that some quinazoline derivatives possessed biological activities, such as antimalarial activity (Lin *et al.*, 2006) antibacterial activity (Bedi *et al.*, 2004) and antifungal activity (Saleh *et al.*, 2004). We report here the crystal structure of 2-(2-chlorophenyl)-2,3-dihydroquinazolin-4(1*H*)-one, (I).

The X-ray crystal structure determination indicates that the pyrimidine ring in the quinazoline moiety is slightly distorted, adopting a skew-boat conformation. The atoms of C2, C3, C8 and N2 are coplanar, with the atoms N1 and C1 deviating from the defined plane by 0.256 (2) and 0.623 (2) Å, respectively. The basal plane is nearly parallel to the benzene ring (C3—C8), forming a dihedral angle of 5.4 (1) °. And is nearly perpendicular to the benzene ring (C9—C14), forming a dihedral angle of 87.7 (1) °. Two benzene rings make a dihedral angle of 87.1 (1) °.

The hydrogen bonds of N—H···O and C—H···N are presented in the crystal structure of (I) (Table 2). The intermolecular hydrogen bond (N1—H1A···O1) and hydrogen bond (C1—H1B···N2) link the adjacent molecules, forming dimmers, respectively. The hydrogen bond of N2—H2A···O1 and above hydrogen bonds link the molecules forming polymers along b (Figure 2).

### Experimental

The title compound, (I), was prepared by the reaction of 2-chlorobenzaldehyde (2 mmol, 0.280 g), 2-aminobenzamide (2 mmol, 0.272 g) and ionic liquid of [Bmim]Br (2 ml) at 353 K. The isolated compound melts at 485–486 K. The single crystals suitable for X-ray diffraction were obtained by slow evaporation ethanol solution.

### Refinement

The H atoms were calculated geometrically and refined as riding, with C—H = 0.93 Å except for H1A, H1B and H2A, and with *U*_iso_(H) = 1.2*U*_eq_.

### Crystal data

**Table:**

C_14_H_11_ClN_2_O	*Z* = 2
*M**_r_* = 258.70	*F*(000) = 268
Triclinic, *P*1	*D*_x_ = 1.399 Mg m^−^^3^
Hall symbol: -P 1	Melting point = 485–486 K
*a* = 6.9900 (1) Å	Mo *K*α radiation, λ = 0.71073 Å
*b* = 8.7488 (2) Å	Cell parameters from 5614 reflections
*c* = 10.4756 (2) Å	θ = 2.4–27.3°
α = 100.639 (1)°	µ = 0.30 mm^−^^1^
β = 92.726 (1)°	*T* = 296 K
γ = 101.786 (1)°	Block, colourless
*V* = 613.91 (2) Å^3^	0.47 × 0.15 × 0.15 mm

### Data collection

**Table:**

Bruker SMART CCD area-detector diffractometer	2204 independent reflections
Radiation source: fine-focus sealed tube	2029 reflections with *I* > 2σ(*I*)
graphite	*R*_int_ = 0.019
φ and ω scans	θ_max_ = 25.2°, θ_min_ = 2.0°
Absorption correction: multi-scan (Jacobson, 1998)	*h* = −8→8
*T*_min_ = 0.901, *T*_max_ = 0.950	*k* = −10→10
8018 measured reflections	*l* = −12→11

### Refinement

**Table:**

Refinement on *F*^2^	Secondary atom site location: difference Fourier map
Least-squares matrix: full	Hydrogen site location: inferred from neighbouring sites
*R*[*F*^2^ > 2σ(*F*^2^)] = 0.030	H atoms treated by a mixture of independent and constrained refinement
*wR*(*F*^2^) = 0.082	*w* = 1/[σ^2^(*F*_o_^2^) + (0.0395*P*)^2^ + 0.1722*P*] where *P* = (*F*_o_^2^ + 2*F*_c_^2^)/3
*S* = 1.07	(Δ/σ)_max_ < 0.001
2204 reflections	Δρ_max_ = 0.20 e Å^−^^3^
176 parameters	Δρ_min_ = −0.19 e Å^−^^3^
0 restraints	Extinction correction: *SHELXL97* (Sheldrick, 2008), Fc^*^=kFc[1+0.001xFc^2^λ^3^/sin(2θ)]^-1/4^
Primary atom site location: structure-invariant direct methods	Extinction coefficient: 0.028 (4)

### Special details

**Table:**

Geometry. All e.s.d.'s (except the e.s.d. in the dihedral angle between two l.s. planes) are estimated using the full covariance matrix. The cell e.s.d.'s are taken into account individually in the estimation of e.s.d.'s in distances, angles and torsion angles; correlations between e.s.d.'s in cell parameters are only used when they are defined by crystal symmetry. An approximate (isotropic) treatment of cell e.s.d.'s is used for estimating e.s.d.'s involving l.s. planes.
Refinement. Refinement of *F*^2^ against ALL reflections. The weighted *R*-factor *wR* and goodness of fit *S* are based on *F*^2^, conventional *R*-factors *R* are based on *F*, with *F* set to zero for negative *F*^2^. The threshold expression of *F*^2^ > σ(*F*^2^) is used only for calculating *R*-factors(gt) *etc*. and is not relevant to the choice of reflections for refinement. *R*-factors based on *F*^2^ are statistically about twice as large as those based on *F*, and *R*- factors based on ALL data will be even larger.

### Fractional atomic coordinates and isotropic or equivalent isotropic displacement parameters (Å^2^)

**Table:**

	*x*	*y*	*z*	*U*_iso_*/*U*_eq_	
Cl1	0.16029 (5)	0.69778 (5)	0.05650 (4)	0.05390 (17)	
N2	0.50252 (18)	1.00296 (13)	0.18929 (11)	0.0344 (3)	
C9	0.53903 (19)	0.72253 (15)	0.14393 (12)	0.0301 (3)	
O1	1.08313 (14)	1.09067 (13)	0.17031 (11)	0.0497 (3)	
C2	0.9066 (2)	1.04662 (16)	0.18541 (14)	0.0370 (3)	
C8	0.61534 (19)	1.08206 (15)	0.30290 (12)	0.0319 (3)	
N1	0.78205 (17)	0.94682 (14)	0.09040 (12)	0.0367 (3)	
C14	0.3498 (2)	0.62845 (16)	0.12560 (13)	0.0345 (3)	
C1	0.57649 (19)	0.88323 (15)	0.10227 (13)	0.0315 (3)	
C10	0.6833 (2)	0.66331 (18)	0.20113 (14)	0.0411 (3)	
H10A	0.8115	0.7230	0.2153	0.049*	
C13	0.3048 (2)	0.48197 (17)	0.16089 (15)	0.0451 (4)	
H13A	0.1769	0.4217	0.1469	0.054*	
C3	0.8198 (2)	1.10531 (17)	0.30508 (13)	0.0384 (3)	
C7	0.5326 (2)	1.14921 (18)	0.41197 (14)	0.0419 (3)	
H7A	0.3970	1.1339	0.4120	0.050*	
C11	0.6398 (3)	0.5163 (2)	0.23777 (16)	0.0521 (4)	
H11A	0.7385	0.4786	0.2765	0.063*	
C4	0.9362 (2)	1.1958 (2)	0.41504 (17)	0.0613 (5)	
H4A	1.0720	1.2122	0.4163	0.074*	
C12	0.4514 (3)	0.42650 (18)	0.21692 (16)	0.0517 (4)	
H12A	0.4232	0.3277	0.2409	0.062*	
C6	0.6509 (3)	1.2379 (2)	0.51937 (16)	0.0582 (5)	
H6A	0.5944	1.2828	0.5916	0.070*	
C5	0.8526 (3)	1.2615 (3)	0.52208 (17)	0.0722 (6)	
H5A	0.9312	1.3214	0.5957	0.087*	
H2A	0.382 (2)	0.9860 (18)	0.1921 (14)	0.037 (4)*	
H1A	0.823 (2)	0.9236 (19)	0.0184 (18)	0.045 (4)*	
H1B	0.510 (2)	0.8682 (16)	0.0185 (14)	0.027 (3)*	

### Atomic displacement parameters (Å^2^)

**Table:**

	*U*^11^	*U*^22^	*U*^33^	*U*^12^	*U*^13^	*U*^23^
Cl1	0.0326 (2)	0.0464 (2)	0.0788 (3)	0.00346 (16)	−0.01097 (18)	0.01200 (19)
N2	0.0259 (6)	0.0347 (6)	0.0423 (7)	0.0069 (5)	0.0038 (5)	0.0067 (5)
C9	0.0304 (7)	0.0324 (6)	0.0270 (6)	0.0067 (5)	0.0065 (5)	0.0041 (5)
O1	0.0298 (5)	0.0554 (7)	0.0566 (7)	−0.0007 (5)	0.0140 (5)	0.0007 (5)
C2	0.0310 (7)	0.0380 (7)	0.0418 (8)	0.0051 (6)	0.0087 (6)	0.0084 (6)
C8	0.0330 (7)	0.0308 (6)	0.0347 (7)	0.0076 (5)	0.0059 (5)	0.0119 (5)
N1	0.0338 (6)	0.0396 (6)	0.0339 (6)	0.0012 (5)	0.0126 (5)	0.0050 (5)
C14	0.0329 (7)	0.0334 (7)	0.0350 (7)	0.0055 (6)	0.0015 (5)	0.0034 (5)
C1	0.0292 (7)	0.0347 (7)	0.0298 (7)	0.0041 (5)	0.0032 (5)	0.0073 (5)
C10	0.0342 (8)	0.0447 (8)	0.0456 (8)	0.0101 (6)	0.0034 (6)	0.0105 (6)
C13	0.0473 (9)	0.0339 (7)	0.0481 (9)	−0.0027 (6)	0.0017 (7)	0.0063 (6)
C3	0.0327 (7)	0.0448 (8)	0.0368 (7)	0.0081 (6)	0.0049 (6)	0.0060 (6)
C7	0.0395 (8)	0.0473 (8)	0.0431 (8)	0.0148 (6)	0.0130 (6)	0.0118 (6)
C11	0.0576 (10)	0.0484 (9)	0.0569 (10)	0.0218 (8)	−0.0019 (8)	0.0175 (7)
C4	0.0382 (9)	0.0858 (13)	0.0502 (10)	0.0114 (9)	−0.0030 (7)	−0.0067 (9)
C12	0.0676 (11)	0.0326 (7)	0.0546 (9)	0.0063 (7)	0.0013 (8)	0.0139 (7)
C6	0.0641 (11)	0.0729 (12)	0.0372 (8)	0.0220 (9)	0.0126 (7)	0.0002 (8)
C5	0.0615 (12)	0.0989 (15)	0.0431 (10)	0.0171 (11)	−0.0070 (8)	−0.0161 (9)

### Geometric parameters (Å, °)

**Table:**

Cl1—C14	1.7453 (14)	C10—C11	1.388 (2)
N2—C8	1.3787 (17)	C10—H10A	0.9300
N2—C1	1.4523 (17)	C13—C12	1.372 (2)
N2—H2A	0.831 (16)	C13—H13A	0.9300
C9—C10	1.3839 (19)	C3—C4	1.388 (2)
C9—C14	1.3915 (19)	C7—C6	1.371 (2)
C9—C1	1.5240 (18)	C7—H7A	0.9300
O1—C2	1.2421 (17)	C11—C12	1.373 (2)
C2—N1	1.3405 (18)	C11—H11A	0.9300
C2—C3	1.4716 (19)	C4—C5	1.375 (2)
C8—C7	1.3924 (19)	C4—H4A	0.9300
C8—C3	1.4001 (19)	C12—H12A	0.9300
N1—C1	1.4511 (17)	C6—C5	1.380 (3)
N1—H1A	0.824 (18)	C6—H6A	0.9300
C14—C13	1.378 (2)	C5—H5A	0.9300
C1—H1B	0.948 (14)		
			
C8—N2—C1	118.48 (11)	C11—C10—H10A	119.5
C8—N2—H2A	116.8 (10)	C12—C13—C14	118.97 (14)
C1—N2—H2A	116.0 (10)	C12—C13—H13A	120.5
C10—C9—C14	116.97 (12)	C14—C13—H13A	120.5
C10—C9—C1	123.74 (12)	C4—C3—C8	119.58 (13)
C14—C9—C1	119.28 (11)	C4—C3—C2	121.32 (14)
O1—C2—N1	121.40 (13)	C8—C3—C2	118.80 (12)
O1—C2—C3	122.54 (13)	C6—C7—C8	120.05 (14)
N1—C2—C3	116.00 (12)	C6—C7—H7A	120.0
N2—C8—C7	121.83 (12)	C8—C7—H7A	120.0
N2—C8—C3	118.83 (12)	C12—C11—C10	120.13 (14)
C7—C8—C3	119.18 (13)	C12—C11—H11A	119.9
C2—N1—C1	124.90 (12)	C10—C11—H11A	119.9
C2—N1—H1A	117.9 (12)	C5—C4—C3	120.63 (16)
C1—N1—H1A	117.1 (12)	C5—C4—H4A	119.7
C13—C14—C9	122.57 (13)	C3—C4—H4A	119.7
C13—C14—Cl1	118.17 (11)	C13—C12—C11	120.28 (14)
C9—C14—Cl1	119.26 (10)	C13—C12—H12A	119.9
N1—C1—N2	108.17 (11)	C11—C12—H12A	119.9
N1—C1—C9	113.41 (11)	C7—C6—C5	121.06 (15)
N2—C1—C9	112.80 (10)	C7—C6—H6A	119.5
N1—C1—H1B	106.9 (8)	C5—C6—H6A	119.5
N2—C1—H1B	107.8 (8)	C4—C5—C6	119.49 (16)
C9—C1—H1B	107.5 (8)	C4—C5—H5A	120.3
C9—C10—C11	121.08 (14)	C6—C5—H5A	120.3
C9—C10—H10A	119.5		
			
C1—N2—C8—C7	−154.80 (12)	Cl1—C14—C13—C12	−179.15 (12)
C1—N2—C8—C3	29.94 (17)	N2—C8—C3—C4	174.78 (14)
O1—C2—N1—C1	176.67 (13)	C7—C8—C3—C4	−0.6 (2)
C3—C2—N1—C1	−5.9 (2)	N2—C8—C3—C2	0.93 (19)
C10—C9—C14—C13	−0.6 (2)	C7—C8—C3—C2	−174.45 (13)
C1—C9—C14—C13	−179.95 (12)	O1—C2—C3—C4	−9.3 (2)
C10—C9—C14—Cl1	178.92 (10)	N1—C2—C3—C4	173.25 (15)
C1—C9—C14—Cl1	−0.48 (16)	O1—C2—C3—C8	164.39 (14)
C2—N1—C1—N2	33.01 (17)	N1—C2—C3—C8	−13.0 (2)
C2—N1—C1—C9	−92.90 (15)	N2—C8—C7—C6	−174.73 (14)
C8—N2—C1—N1	−44.49 (15)	C3—C8—C7—C6	0.5 (2)
C8—N2—C1—C9	81.78 (14)	C9—C10—C11—C12	0.3 (2)
C10—C9—C1—N1	17.84 (18)	C8—C3—C4—C5	0.6 (3)
C14—C9—C1—N1	−162.81 (11)	C2—C3—C4—C5	174.27 (18)
C10—C9—C1—N2	−105.57 (14)	C14—C13—C12—C11	0.3 (2)
C14—C9—C1—N2	73.78 (15)	C10—C11—C12—C13	−0.6 (3)
C14—C9—C10—C11	0.2 (2)	C8—C7—C6—C5	−0.4 (3)
C1—C9—C10—C11	179.58 (13)	C3—C4—C5—C6	−0.5 (3)
C9—C14—C13—C12	0.3 (2)	C7—C6—C5—C4	0.4 (3)

### Hydrogen-bond geometry (Å, °)

**Table:**

*D*—H···*A*	*D*—H	H···*A*	*D*···*A*	*D*—H···*A*
N2—H2A···O1^i^	0.831 (16)	2.461 (16)	3.1847 (16)	146.2 (14)
N1—H1A···O1^ii^	0.824 (18)	2.103 (18)	2.9146 (16)	168.4 (16)
C1—H1B···N2^iii^	0.948 (14)	2.635 (14)	3.4369 (17)	142.6 (11)

Symmetry codes: (i) *x*−1, *y*, *z*; (ii) −*x*+2, −*y*+2, −*z*; (iii) −*x*+1, −*y*+2, −*z*.

## References

[bb1] Bedi, P. M. S., Kumar, V. & Mahajan, M. P. (2004). *Bioorg. Med. Chem. Lett.* **14**, 5211–5213. 10.1016/j.bmcl.2004.07.065 15380229

[bb2] Bruker (2001). *SAINT* and *SMART* Bruker AXS Inc., Madison, Wisconsin, USA.

[bb3] Feng, Z., Chen, X., Guo, Z., Jiang, Y., Li, J., Zhu, F., Guo, Y., Li, Y. & Fu, J. (2006). Chinese Patent CN 1854130 A, 1 Nov 2006.

[bb5] Jacobson, R. (1998). Private communication to the Rigaku Corporation, Tokyo, Japan.

[bb6] Keenan, T. P. & Shakespear, W. C. (2004). PCT Int. Appl. WO 2004058267 A1, 15 Jul 2004.

[bb7] Lin, A. J., Guan, J., Zhang, Q. & Skillman, D. R. (2006). US Patent Appl. Publ. US 2006094736 A1, 4 May 2006.

[bb8] Mikiciuk-Olasik, E., Blaszczak-Swiatkiewiz, K., Zurek, E., Krajewska, U., Rozalski, M., Kruszynski, R. & Bartczak, T. J. (2004). *Arch. Pharm.* **337**, 239–246. 10.1002/ardp.200100656 15095417

[bb9] Saleh, M. A., Hafez, Y. A., Abdel-Hay, F. E. & Gad, W. I. (2004). *Phosphorus Sulfur Silicon Relat. Elem.* **179**, 411–426.

[bb10] Sheldrick, G. M. (2008). *Acta Cryst.* A**64**, 112–122. 10.1107/S0108767307043930 18156677

